# Outcome of Patients with Primary Immune-Complex Type Mesangiocapillary Glomerulonephritis (MCGN) in Cape Town South Africa

**DOI:** 10.1371/journal.pone.0113302

**Published:** 2014-11-20

**Authors:** Ikechi G. Okpechi, Thandiwe A. L. Dlamini, Maureen Duffield, Brian L. Rayner, George Moturi, Charles R. Swanepoel

**Affiliations:** 1 Division of Nephrology and Hypertension, Groote Schuur Hospital and University of Cape Town, South Africa; 2 Division of Anatomical Pathology, National Health and Laboratory Services (NHLS), University of Cape Town, South Africa; 3 Department of Medicine, Aga Khan University Hospital, Nairobi, Kenya; Mario Negri Institute for Pharmacological Research and Azienda Ospedaliera Ospedali Riuniti di Bergamo, Italy

## Abstract

**Background and Aim:**

Mesangiocapillary glomerulonephritis (MCGN) is a common cause of chronic kidney disease in developing countries. Data on the renal outcome of patients with idiopathic MCGN is limited. The aim of this study is to investigate the outcome of patients with idiopathic MCGN presenting to the Groote Schuur Hospital (GSH) Renal Unit in Cape Town.

**Materials and Methods:**

A retrospective study of patients with idiopathic MCGN followed up at our clinic. Seventy-nine patients with no identifiable cause of MCGN were included for analysis. A composite renal outcome of persistent doubling of serum creatinine or end stage renal disease (ESRD) was used. Kaplan Meier survival and Cox regression analysis were used to assess survival and identify factors predicting the outcome.

**Results:**

The mean age at biopsy was 33.9±13.6 years and 41.8% were black. Mean duration of follow up was 13.5±18.8 months. Twenty-three patients (34.2%) reached the composite endpoint. Overall, median renal survival was 38.7±11.7 months (95% CI 15.7–61.8) with 2-year and 5-year renal survival of 61% and 40.3% respectively. No significant difference was found for renal survival between males and females, treatment or non-treatment with immunosuppression, presence or absence of crescents or histological type of MCGN (p>0.05). On univariate Cox-regression analysis, factors found to be associated with the outcome were the estimated glomerular filtration rate at biopsy (OR 0.97 [95%CI: 0.95–0.99], p<0.0001), black race (OR 3.03 [95%CI: 1.27–7.21], p = 0.012) and presence of interstitial fibrosis in the biopsy (OR 2.64 [95%CI: 1.07–6.48], p = 0.034). Age, systolic blood pressure and attaining complete or partial remission approached significant values with the endpoint.

**Conclusions:**

The outcome of idiopathic MCGN in Cape Town is poor and requires further prospective studies to improve our understanding of this common disease.

## Introduction

Mesangiocapillary glomerulonephritis (MCGN; also known as membranoproliferative GN [MPGN]) is a histological pattern of glomerular injury characterized by mesangial hypercellularity, increased mesangial matrix and thickening of glomerular capillary walls secondary to subendothelial deposition of immune complexes and/or complement factors, cellular entrapment, and new basement membrane formation [1], [2]. MCGN has traditionally been divided into three distinct morphological types: type I (classical MCGN), is characterized by the presence of subendothelial deposits of immune complexes; type II MCGN (dense deposit disease), characterized by the presence of dense deposits in the basement membrane and type III MCGN, (considered as a variant of type I) and characterized by the presence of additional subepithelial deposits.

MCGN is a common cause of glomerulonephritis and the nephrotic syndrome in many low to middle income countries but especially in Africa [3]–[8]. In Romania, MCGN was the most frequent primary glomerulonephritis (GN) and was responsible for 29.4% of all primary glomerulonephritides reported from 1995 to 2004 [3]. In a previous study from our centre, we reported MCGN to account for 20.4% of all primary GN with 90.4% of cases being type I MCGN [6]. However, IgA nephropathy remains the most common primary glomerular disease reported from many developed countries where the occurrence of primary MCGN has steadily declined in recent decades [9]–[12]. Although the “hygiene hypothesis” [13], [14] may explain some of the differences in prevalence of glomerular diseases seen in emerging and developed countries, results from recent research in this field has now made some authors to question the existence of idiopathic MCGN [15]. Their doubt is predominantly borne out of advances in methods of analysis of biopsy specimens and a more thorough and detailed evaluation of patients to identify possible causes of so-called idiopathic MCGN [15]. However, these studies have been published from high income countries where IgAN is still predominant. Sethi et al have therefore proposed a new classification for MCGN based on immune complex deposition (with or without complement) and sole complement deposition in the glomerulus denoting dysregulation of the alternative pathway of complement [16] (see Figure S1 and Figure 2).

The treatment recommendation of the KDIGO on the use of immunotherapies in idiopathic MCGN is only limited to cases in which crescents are present [17] and treatment of adults with the disease is often unrewarding as approximately 60% of patients will progress to end-stage renal disease (ESRD) within 10 years [18]–[20]. Given that so-called idiopathic MCGN is the most frequent primary GN seen in our population, the aim of this study is to report on the outcome of patients in Cape Town with idiopathic MCGN and to identify the factors that predict renal outcomes in such patients who are longitudinally followed up in our centre.

## Materials and Methods

### Ethics Statement

The study received approval from the Human Research Ethics Committee (HREC REF: 227/2012) of the University of Cape Town. All patient records/information was anonymized and de-identified prior to analysis.

### Study population

Hospital records of patients who had a kidney biopsy performed between January 2000 to December 2011 and who were diagnosed with idiopathic MCGN and followed up at the renal clinic at Groote Schuur Hospital Cape Town were retrieved for retrospective collection of data. The written records and the electronic records of these patients were rigorously assessed in order to exclude patients with MCGN with possible secondary cause. All selected patients were proven to have tested negative for common viral infections (HIV, Hepatitis B and C), other commonly encountered infections (such as mycobacterium tuberculosis, syphilis) and autoimmune diseases (lupus and rheumatoid arthritis). As these patients were not acutely ill at presentation or showing evidence for any chronic infections, tests for malaria, schistosomiasis, leprosy among other possible infections that have been reported to be associated with MCGN were not performed; and although these infections are unusual in Cape Town, the patients never manifested any features of them during follow up. Serum protein electrophoresis, urine protein electrophoresis and bone marrow biopsy were not clinically indicated and were thus not performed in our patients. We identified 85 patients with complete records and with no known secondary causes of MCGN but excluded 6 patients whose biopsies only had C3 complement deposits alone.

### Data Collection

Demographic, clinical and biochemical records of patients included in the study were obtained at the time of biopsy and during follow-up visits to the renal clinic. Data collected therefore included age at time of biopsy, gender, indication for biopsy, duration of follow up, serial recorded blood pressures (systolic and diastolic) and various laboratory measurements (serum albumin, creatinine, cholesterol, complements (C3 and C4 – expressed as normal or low) and proteinuria [g/24 hrs]. The racial grouping of our patient population was categorized as Black Africans and non-Black Africans (to include patients of mixed ancestry and Whites). The estimated glomerular filtration rate was calculated using the Modification of Diet in Renal Disease (MDRD) formula [21]. Treatment received by the patients was also recorded.

### Histology

Light microscopic and immunohistochemical features of the renal biopsies were noted and recorded. Histological data collected included number of glomeruli per biopsy, percentage of sclerosed glomeruli reported, degree of interstitial fibrosis (none, mild, moderate or severe), presence of crescentic lesions and type of deposit present on immunohistochemical analysis (IgA, IgG, IgM or C3). All patients included had immune complex type MCGN (immune complex deposits were present with or without complement deposit) according to the newly proposed classification of Sethi et al [16]. Those with only C3 deposits were excluded in the analysis. Electron microscopic examination of all biopsy specimens was performed. All the histological specimens were reviewed by one pathologist (M.D.).

### Definitions used in this study

#### Blood pressure

Hypertension was defined as systolic BP (SBP) persistently ≥140 mmHg and/or diastolic BP (DBP) ≥90 mmHg or if on treatment with anti-hypertensive medications [22]. Blood pressure at biopsy and average BP during follow up visits were recorded.

#### Remission

As there is no guideline for defining remission in MCGN, we used the following criteria and categorized patients into 3 groups: complete remission (CR), partial remission (PR) or no remission (NR) [23]:

- CR was defined as proteinuria of <0.2 g/day with stable eGFR if normal at baseline or increase in eGFR by 25% if abnormal at baseline.
- PR was defined as reduction in proteinuria (for proteinuria between 0.2 and 2.9 g) and stable eGFR if normal at baseline or increase in eGFR by 25% if abnormal at baseline.
- NR was defined as persistent proteinuria of ≥3 g/day or progressive or worsening renal impairment.

### Study end-point

The composite end point of this study was persistent doubling of the serum creatinine over the baseline value or end-stage renal disease (ESRD). For patients who reached the end point, the period of follow-up was the interval between first renal biopsy and the time the end point was reached.

### Statistics

The data were analyzed using IBM SPSS Statistics 21 software (SPSS, Chicago, IL). Categorical variables were presented as percentages and continuous variables as means ± SD. Comparison was made between those reaching the end-point and those not reaching the end-point using the Student's t-test, chi-square test or Fisher's exact test. Estimate of survival was done using the Kaplan–Meier survival method. Renal survival with time to ESRD was assessed using Kaplan–Meier estimates and log-rank test for comparison of survival estimates between groups. Univariate analysis was performed using Cox regression analysis to assess the association between relapse-free survival and explanatory variables. Significant P-value was taken as P<0.05.

## Results

### Baseline characteristics of the patients

There were a total of 79 patients eligible for inclusion in the study with renal biopsy diagnosis of MCGN and with no clinical or biochemical evidence for a secondary cause of MCGN. The mean age of all the patients was 33.9±13.6 years with 74.7% of the patients being males. Racial distribution of the subjects was 41.8%, 54.4% and 3.8% for blacks, patients of mixed ancestry and whites respectively. We observed that 20.3% of the patients were either actively abusing substance (predominantly methamphetamines – called “*Tik*” in Cape Town) or were referred to us from a correctional services department for treatment. Nephrotic syndrome was by far the most frequent indication for renal biopsy in 77.9%. Other features at time of biopsy are shown in Table 1.

**Table 1:** Demographic, clinical and biochemical features of all the patients.

Baseline characteristics (n = 79)	Value
Mean age (years)	33.9±13.6
Gender (Male) (%)	74.7
Ethnicity (%):	
- Blacks	41.8
- Mixed ancestry	54.4
- Whites	3.8
Mean duration of follow-up (months)	13.5±18.8
Hypertension at biopsy (%)	65.8
Oedema present at biopsy (%)	84.8
History of substance abuse or incarceration (%)	20.3
History of schizophrenia (%)	5.1
SBP at biopsy (mmHg)	159.9±30.1
DBP at biopsy (mmHg)	95.8±17.8
Indication for renal biopsy (%):	
- Nephrotic syndrome	77.9
- Nephrotic-nephritic syndrome	10.3
- AKI	5.9
Serum albumin at biopsy (g/L)	25.9±7.3
Serum cholesterol at biopsy (mmol/L)	6.7±2.3
Serum creatinine at biopsy (µmol/L)	180.6±166.8
Estimated MDRD GFR at biopsy (ml/min/1.73 m^2^)	65.1±35.2
Proteinuria at biopsy (g/24 hrs)	8.2±6.4
Low complement C3 at biopsy (%)	15.2
Low complement C4 at biopsy (%)	-

SBP – Systolic blood pressure, DBP – Diastolic blood pressure, AKI – Acute kidney injury, MDRD – Modification of diet in renal failure, GFR – Glomerular filtration rate.

### Histological characteristics of the renal biopsies

Table 2 summarizes the histological features of the biopsies. The average number of glomeruli per biopsy was 16.5±10.5 with an average of 6.2% glomeruli reported as sclerosed. In 63.5% the interstitium was completely normal with no evidence of fibrosis at the time of biopsy; severe interstitial fibrosis was present in 4.1%. IgM deposits (59.4%) and C3 deposits (69.8%) were more frequently seen.

**Table 2:** Histological features of the renal biopsies (n = 79).

Variable	Value
Number of glomeruli	16.5±10.5
Interstitial fibrosis (%):	
- No fibrosis	63.5
- Mild fibrosis	24.3
- Moderate fibrosis	8.1
- Severe fibrosis	4.1
Mean percentage of sclerosed glomeruli (%)	6.2
Crescentic lesions present (%)	17.1
Immunohistochemical features (%):	
- IgA deposit present	18.8
- IgG deposit present	43.8
- IgM deposit present	59.4
- C3 deposit present	69.8

Ig – Immunoglobulin, C3 – Complement 3.

### Comparison of the features of patients reaching or not reaching end-point

Twenty-three of 79 patients (34.2%) reached the end-point. There were more black African patients reaching end-point than non-black patients (60.9% vs 33.9%; p = 0.044). Average of all systolic and diastolic blood pressures during follow up visits were significantly higher in those who reached the end-point than in those who did not reach the end-point (p<0.05) (Table 3). However, SBP and DBP at initial presentation, although higher in those reaching the end-point were not significantly different. A significantly higher proportion of patients had attained a complete or partial remission at six months after diagnosis in those not reaching end-point than in those who did (36.2% vs 4.8%; p = 0.007).

**Table 3:** Comparison of clinical and histological factors associated with outcome.

Factors	End-point		p
	NO (n = 56)	YES (n = 23)	
Age at biopsy (years)	33.0±12.6	36.1±16.0	0.364
Duration of follow-up (Months)	11.6±17.1	17.9±22.1	0.178
Gender (Male) (%)	71.4	82.6	0.398
Race (%):			**0.044**
- Blacks	33.9	60.9	
- Non-Blacks	66.1	39.1	
Hypertension at biopsy (%)	64.3	69.6	0.796
SBP at initial presentation (mmHg)	155.3±29.4	167.9±30.6	0.198
Average SBP at follow-up (mmHg)	141.7±19.8	168.1±30.1	**0.003**
DBP at initial presentation (mmHg)	93.4±18.5	100.0±16.3	0.257
Average DBP at follow-up (mmHg)	88.4±13.8	99.2±19.2	**0.018**
Complete or partial remission at 6 months:	36.2	4.8	**0.007**
Histological features (%):			
- IgA deposits present	22.7	21.1	1.000
- IgG deposits present	47.7	57.9	0.585
- IgM deposits present	68.2	68.4	1.000
- C3 deposits present	62.8	78.9	0.252
- Any interstitial fibrosis present (%)	30.4	56.5	**0.041**
- Any crescentic lesion present (%)	14.3	26.1	0.330

SBP – Systolic blood pressure, DBP – Diastolic blood pressure, Ig – Immunoglobulin, C3 – Complement 3.

The frequencies of immune (IgA, IgG and IgM) or complement (C3) deposits observed in the biopsies were not significantly different in both groups. Presence of any interstitial fibrosis (mild, moderate or severe) was significantly higher in the group reaching end-point (56.5% vs 30.4%; p = 0.041); although there were more patients in the group that reached end-point with crescentic lesion present on biopsy, this was not significantly different between the 2 groups. Of all the biochemical features, only serum creatinine (and estimated GFR) at presentation were significantly different between the 2 groups (Table 4). Those who reached the end-point had a significantly higher value of serum creatinine (p = 0.001) at the time of renal biopsy (which was usually the first presentation of the patient). There were more patients in the end-point group with low serum complement; however, this was not significantly different from those not reaching the end-point.

**Table 4:** Comparison of biochemical and treatment factors associated with outcome.

Factors	End-point		p
	NO (n = 56)	YES (n = 23)	
Complement C3 at biopsy	1.05±0.41	1.23±0.62	0.311
Low complement C3 at biopsy (%)	14.3	17.4	0.706
Complement C4 at biopsy	0.36±0.33	0.37±0.19	0.891
Low complement C4 at biopsy (%)	-	-	-
Serum cholesterol (mmol/L)	6.1±1.4	7.9±3.2	0.079
Serum albumin at biopsy (g/L)	26.1±6.9	25.2±8.2	0.667
Serum creatinine at biopsy (µmol/L)	124.7±86.9	315.2±229.0	**0.001**
Estimated MDRD GFR at biopsy (ml/min)	75.3±30.6	40.5±34.0	**<0.0001**
Proteinuria at biopsy (g/day)	8.1±6.9	8.3±5.2	0.872
Treatment with ACE-i/ARB (%)	92.9	87.0	0.409
Treatment with prednisone (%)	25.0	52.2	**0.034**
Treatment with cyclophosphamide (%)	8.9	26.1	0.071
Treatment with Azathioprine (%)	3.6	8.7	0.635

MDRD – Modification of Diet in Renal Disease; GFR – Glomerular filtration rate; ACE-I – Angiotensin converting enzyme inhibitors; ARB – Angiotensin receptor blockers.

As treatment often followed a conservative care approach, use of an angiotensin converting enzyme inhibitor (ACE-i) or angiotensin receptor blocker (ARB) was common but was not significantly different between the 2 groups (87.0% vs 92.9%; p = 0.409). There were more patients who reached endpoint that received immunosuppression therapy: prednisone: 52.2% vs 25.0%; p = 0.034, pulse cyclophosphamide: 26.1% vs 8.9%; p = 0.071, Azathioprine: 8.7% vs 3.6%; p = 0.635).

### Survival Analysis

**Figure 1:**
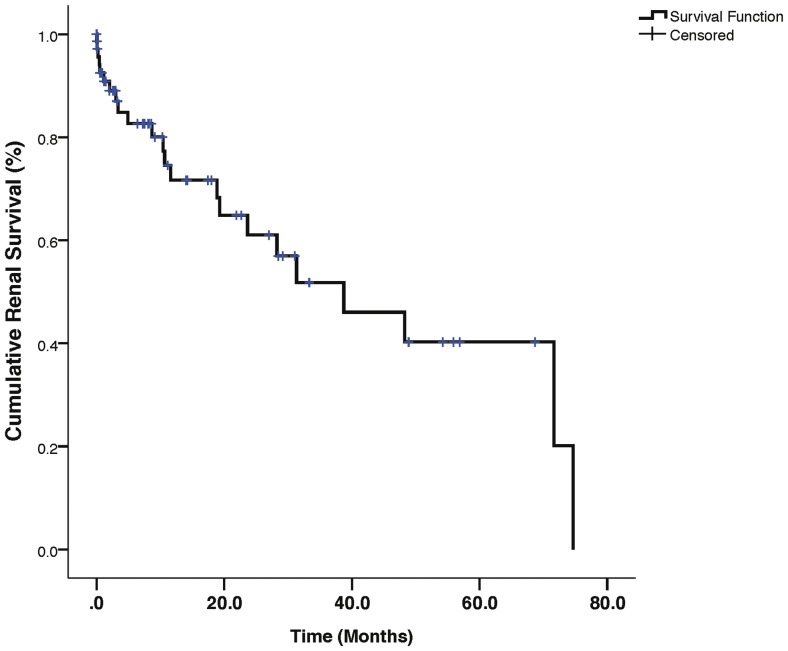
Kaplan-Meier curve for overall renal survival in the study population.

**Figure 2:**
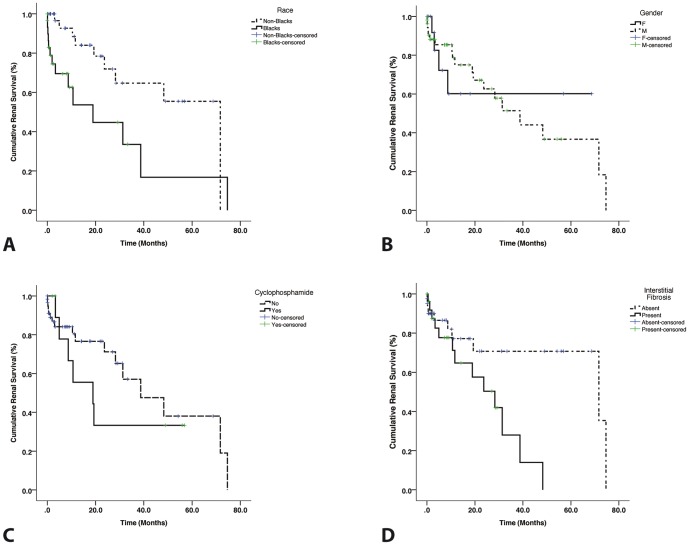
Kaplan-Meier curve for renal survival based on histological features of MCGN. (A) shows differences in outcome between Black Africans and non-Black Africans (log rank p = 0.009); (B) shows gender differences in outcome (log rank p = 0.995); (C) differences between patients who received treatment with cyclophosphamide and those who didn't receive treatment (log rank p = 0.440) and (D) shows differences in outcomes based on the presence of interstitial fibrosis in the biopsy (log rank p = 0.028).

The cumulative renal survival curve is shown in Figure 1. Overall, the median renal survival was 38.7±11.7 months (95% confidence interval [95%CI]: 15.7–61.8) with 2-year and 5-year renal survival being 61.0% and 40.3% respectively. Kaplan-Meier renal survival curves for differences in outcome based on race (log rank p = 0.009), gender (log rank p = 0.995), treatment with I/V cyclophosphamide (log rank p = 0.440) and presence or absence of interstitial fibrosis (log rank p = 0.028) are shown in figure 2. *Cox univariate regression analysis for predictors of end-point*.

Factors associated with the end-point, identified through Cox-regression analysis are shown in Table 5. Estimated GFR at biopsy (OR 0.97 [95%CI 0.95–0.99], p<0.0001), being of black African descent (OR 3.03 [95%CI 1.27–7.21], p = 0.012) and presence of interstitial fibrosis at biopsy (OR 2.64 [95%CI 1.07–6.48], p = 0.034) were the factors identified to be associated with the end-point. Early complete or partial remission (at six months after renal biopsy) did not influence the renal outcome in these patients (OR: 0.17 [95%CI: 0.02–1.27]; p = 0.084).

**Table 5:** Cox univariate regression analysis for predictors of the end-point.

Variable	OR (95% CI)	P value
Age	1.03 (0.99–1.06)	0.089
Gender (Female)	0.99 (0.33–2.99)	0.995
Estimated GFR at biopsy	0.97 (0.95–0.99)	**<0.0001**
Average SBP during follow-up	1.01 (0.99–1.03)	0.082
Average DBP during follow-up	1.00 (0.98–1.03)	0.982
Remission status at 6 months (CR/PR)	0.17 (0.02–1.27)	0.084
Race (Blacks)	3.03 (1.27–7.21)	**0.012**
Interstitial fibrosis (Present)	2.64 (1.07–6.48)	**0.034**
Crescent (Present)	1.66 (0.64–4.29)	0.295
Cyclophosphamide (Yes)	1.45 (0.56–3.78)	0.443

GFR – Glomerular filtration rate; SBP – systolic blood pressure; DBP – diastolic blood pressure; CR – complete remission, PR – partial remission.

## Discussion

The analysis of our data of patients with idiopathic MCGN in Cape Town shows that a number of important demographic, clinical and histological features may adversely predict the renal outcome in these patients. Given that there are no published recent outcome studies on idiopathic MCGN, this study has shown that in comparison with previous studies, the renal outcome of patients with idiopathic MCGN remains dismally low in comparison to outcomes in previously published studies (Table 6) [18]–[20], [24]–[28]. Importantly, we have observed that lower estimated GFR at presentation (often a surrogate of late presentation), being of black African ethnicity and presence of interstitial fibrosis in the renal histology were the predicting factors of renal outcome in our patients. The 2-year and 5-year renal survival was found to be low in this study.

**Table 6:** Summary of some selected studies reporting outcomes in idiopathic MCGN.

Author (Year publication) [REF]	Sample size	Country of study	Study design	Mean duration of follow-up (years)	Study population	Renal survival	Predictors of outcome
Habib R (1973) [18]	105	France	Retrospective	5.75	Paediatric	Type I: 66% Type II: 45%	Crescents, Type II disease, nephrotic syndrome, macroscopic haematuria, impaired renal function at presentation
Swainson et al (1983) [24]	40	UK	Observational	5–22	Adult and Paediatric	5-year: 58% 10-year: 48%	Low complements, crescents, impaired renal function at presentation
Cameron et al (1983) [20]	104	UK	Observational	8	Adult and paediatric	Type I: 62% Type II: 51%	Glomerulosclerosis, crescents, nephrotic syndrome, Type II disease
McEnery (1990) [25]	76	USA	Retrospective	10.6	Paediatric	10-year: 82% 20-year: 56%	Not analyzed
Orlowski et al (1988) [26]	50	Poland	Observational	10	Adult	5-year: 90% 10-year: 82%	Hypertension
Schmitt et al (1990) [19]	220	Germany	Retrospective	5	Adult and Paediatric	5-year: 51% 10-year: 36%	Hypertension, crescents, interstitial fibrosis
Perdersen (1995) [27]	37	Denmark	Observational	2.7	Adult and Paediatric	5-year: 35% 10-year: 16%	Older age, hypertension
Little et al (2006) [28]	70	UK	Retrospective	13.8	Adult and paediatric	8.3-20 years: 30%*	Nephrotic range proteinuria, crescents, mesangial proliferation
This study	79	South Africa	Retrospective	1.1	Adult	2-year: 61.0% 5-year: 40.3%	Abnormal serum creatinine at presentation, black race, interstitial fibrosis, hypertension

UK – United Kingdom; USA – United States of America.

*Median time to ESRD.

Late presentation of patients is common in many centres in Africa and is thought to be an important factor responsible for disease outcome [7], [29], [30]. Many late presenters will have markedly elevated serum creatinine (low eGFR) and may have features of ureamia or be in need of dialysis at first presentation. Late presenters may account for 36.5% of our patients having significant interstitial fibrosis on histology (Table 2). Reasons for late presentation are often tied to poverty (lack of transportation, long distances to health care facility, lack of health insurance, low level of education), cultural beliefs (visits to the traditional healer) or due to late referral. Although in this study we did not evaluate the duration of symptoms in our patients, many patients often report having been ill for many weeks and in some instances many months before presenting to hospital. In one study, late evaluation of patients with chronic kidney disease (CKD) was reported to be associated with greater burden and severity of comorbid disease, black ethnicity, lack of health insurance, and shorter duration of survival [31]. However, late presentation alone may not explain the elevated serum creatinine as the pathogenesis of MCGN itself (involving complement activation, capillary wall damage and reduction in filtration at the glomerulus) may have accounted for this. We did not exclude the possible effects of genetic factors relative to outcome in this study; hence our finding of the black race being associated with outcome may be put down to socio-economic factors rather than genetic as many of the black patients who use the public health care system in South Africa are indigent.

Although SBP and DBP during follow-up were not predictors of the endpoint on regression analysis, they were significantly higher in those patients reaching endpoint than those who didn't. Uncontrolled hypertension combined with impaired sieving function with consequent protein overload play a pathogenic role in the progression of CKD. The utility of adequate BP control to reduce progression of kidney disease in diabetic and non-diabetic CKD has been shown from various studies and is recommended by various guidelines [21], [22], [32], [33]. One systematic review and meta-analysis of randomized controlled trials on the effects of intensive BP lowering on the progression of CKD has reported that in 5 trials that involved 1703 patients, intensive BP lowering reduced the risk of progressive kidney failure by 27% in people with proteinuria at baseline [34]. In one large population based study, the odds ratios to develop progression of urine albumin excretion during follow-up was 1.91 (95% CI 1.72 to 2.12) per 10-mmHg increase in BP during follow-up and this was independent of baseline BP and other biochemical and patient factors during follow-up [35]. In our study, several patients were receiving treatment with an agent that blocked the renin angiotensin aldosterone pathway. Previous studies from our centre have also highlighted inadequate control of BP as a factor for poor outcomes in patients with proliferative and non-proliferative glomerulonephritis [36], [37]. Poor BP control may be related to the disease process or in some instances to poor adherence to therapy. Emphasis on BP control in patients with MCGN may therefore be useful in improving renal outcomes since, unlike in many other glomerular diseases, immunosuppression has a limited role in the treatment of patients.

Renal survival in MCGN has often been reported to be low and with probably worse outcomes in comparison with other glomerular diseases, probably due to lack of specific therapy for MCGN. Recommendations from the KDIGO guideline for treatment of MCGN are limited to cases with abnormal renal function and are drawn mainly from small observational studies [17]. It was interesting that treatment with immunosuppression did not make any difference with regards to the outcome even though there were more patients in our study who reached endpoint that received immunosuppression (Table 4). Renal survival in this study was found to be 61% at 2 years and 40.3% at 5 years, much lower than for studies reported from developed countries. Although some older studies have found type of MCGN to predict outcome, we did not apply this to our study given the new approach to classifying patients with MCGN [16]. However, like some previous studies have reported, we found that any degree of interstitial fibrosis present at time of biopsy (mild/moderate/severe) is a poor prognostic factor for renal outcome.

Given recent publications on this subject, a major question that this study needed to address is whether everything was done to exclude all known secondary causes of MCGN before a labeling of the “idiopathic” diagnosis. The answer to that question is “yes – as far as was possible” given the clinical features of the patients at time of presentation and during follow up. All the patients included in this study had the immune-complex type of MCGN hence, secondary causes will include various chronic bacterial and viral infections, autoimmune diseases and monoclonal gammopathy (dysproteinaemia). All the patients included in this study tested negative for HIV, hepatitis B and C, syphilis and did not demonstrate serological or histological features of post-infectious glomerulonephritis. Our patients also tested negative for cryoglobulins and commonly occurring autoimmune diseases like SLE and rheumatoid arthritis. As it was not clinically indicated, no patient was tested for a dysproteinaemia and there were no features to suggest the disease being present at time of presentation or during the period of follow-up. Our patients typically present at a younger age (mean age in this study is 33.9±13.9 years) compared to other studies where dysproteinaemias have been reported [38], [39]. The lack of evidence for a secondary cause of MCGN is supported by the absence of clinical manifestation of any chronic infection or systemic disease during the period of follow up in our patients. However, the high frequency of patients with IgM deposits (59.4%) in their biopsy may be suggestive of a recent (maybe sub-clinical infection) at time of biopsy.

There still remains on our part a persistent and a renewed concern that there is a huge gap in our understanding of this common disease in Cape Town even though we continue to see many “healthy” patients presenting with nephrotic syndrome in whom renal histology show MCGN that is labeled as idiopathic after a detailed and thorough assessment. It could be that we have continued to miss a yet to be identified sub-clinical chronic antigenaemic process possibly resulting from an environmental exposure to drugs (substance abuse), tattoo ink or previous incarceration from one of the correctional services department in Cape Town all of which we have observed to be quite common amongst these patients. Further prospective studies are required to reprove this.

There were a number of limitations for this study. Firstly is our inability to assess for the monoclonality of the Ig deposits at time of biopsy. However, as our patients were young and never exhibited clinical features to suggest monoclonal gammopathy at any time during follow-up, this diagnosis was clinically excluded. Secondly, given the high number of patients with intravenous drug abuse and the common finding of IgM deposition in glomeruli, our study is also limited by the absence of routine testing for IgG/IgM mixed cryoglobulins. However, given that cryoprecipitates which can occasionally be observed as hyaline-like globules were not observed in the histological materials of these patients further makes this unlikely. Finally, the retrospective design of this study limited the type of data that can be collected and analyzed. This therefore warrants a prospective study of patients with “idiopathic” MCGN with emphasis on a more rigorous evaluation to exclude or identify possible secondary causes. For now however, patients identified with MCGN in whom we are unable to find a cause, efforts to reduce proteinuria and treat BP to target will be our major aim.

## Conclusions

The outcome of idiopathic MCGN in Cape Town is poor and is related to socio-demographic as well as to some clinical and histological features at time of presentation. Being the most frequent primary glomerular disease reported in Cape Town, there is need for a prospectively designed study of idiopathic MCGN in order to increase our understanding of its pathogenesis and maybe find ways to its treatment.

## Supporting Information

Figure S1 **Glomerular features of one of our study patients identified with immune complex type MCGN.** A – The H&E stain showing increased lobulation of the displayed glomerulus and increased mesangial matrix; B – silver stain showing double contours/splitting of the glomerular basement membrane; C – F shows positive immunohistochemical stains for C3, IgG, IgA and IgM respectively; G–I are the electron micrographs (x 30,000) showing sub-endothelial and intramembranous deposits. (Courtesy Dr M Duffield and Mr. D. Rademeyer – National Health and Laboratory Services [NHLS] Cape Town).
(TIFF)
