# Supplementary figures and images for: Outcome of Patients with Primary Immune-Complex Type Mesangiocapillary Glomerulonephritis (MCGN) in Cape Town South Africa
Source: PLoS One. 2014 Nov 20;9(11):e113302. doi: 10.1371/journal.pone.0113302 (PMC4239048; doi:10.1371/journal.pone.0113302)

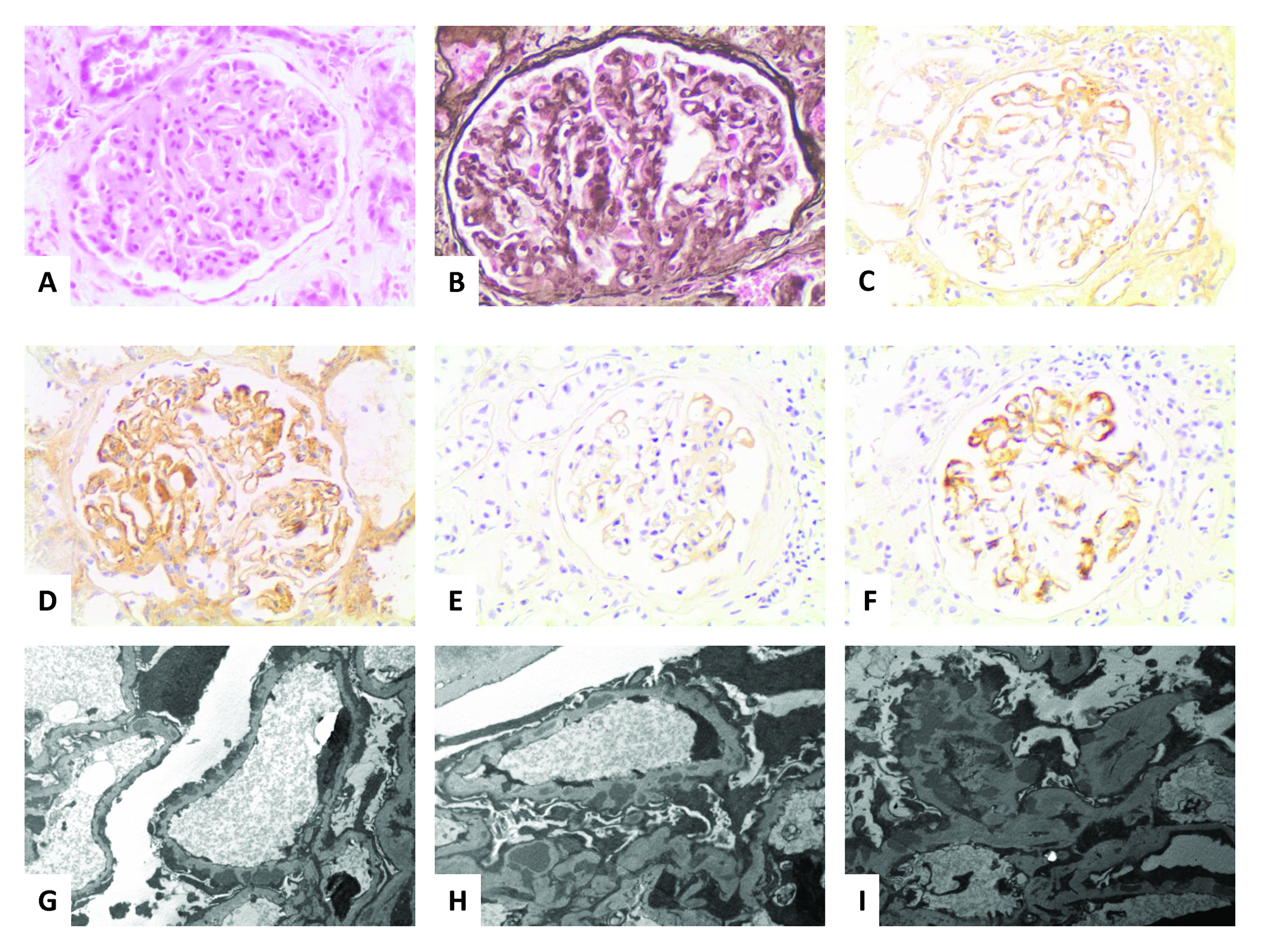

Supplement: Figure S1 — Glomerular features of one of our study patients identified with immune complex type MCGN. A – The H&E stain showing increased lobulation of the displayed glomerulus and increased mesangial matrix; B – silver stain showing double contours/splitting of the glomerular basement membrane; C – F shows positive immunohistochemical stains for C3, IgG, IgA and IgM respectively; G–I are the electron micrographs (x 30,000) showing sub-endothelial and intramembranous deposits. (Courtesy Dr M Duffield and Mr. D. Rademeyer – National Health and Laboratory Services [NHLS] Cape Town). (TIFF) [file pone.0113302.s001.tiff]
